# Supplementary material for: Redistribution of PU.1 partner transcription factor RUNX1 binding secures cell survival during leukemogenesis
Source: EMBO J. 2024 Nov 14;43(24):6291–309. doi: 10.1038/s44318-024-00295-y (PMC11649769; doi:10.1038/s44318-024-00295-y)
Supplement: Supplementary file 3 — Appendix [file 44318_2024_295_MOESM3_ESM.pdf]

# APPENDIX FOR:

## Redistribution of PU.1 partner transcription factor RUNX1 binding secures cell survival during leukemogenesis

Alexander Bender, Füsun Boydere, Ashok Kumar Jayavelu, Alessia Tibello, Thorsten König, Hanna Aleth, Gerd Meyer zu Hörste, Thomas Vogl, and Frank Rosenbauer

**Table of Contents:**

Appendix Material & Methods (pages 2-7)

Appendix Figures S1-S5 (pages 8-15)

Appendix References (page 16)

## Appendix Material & Methods

### Flow Cytometry Antibodies

| Marker | Fluorochrome/Channel | Company/ID |
|---|---|---|
| Cd3 | PerCP-Cy5.5 | BioLegend 100328 |
| Cd4 | PerCP-Cy5.5 | BioLegend 100434 |
| Cd8 | PerCP-Cy5.5 | BioLegend 100734 |
| Cd19 | PerCP-Cy5.5 | BioLegend 115534 |
| Cd45r | PerCP-Cy5.5 | BioLegend 103236 |
| Gr1 | PerCP-Cy5.5 | BioLegend 108427 |
| Ter119 | PerCP-Cy5.5 | BioLegend 116228 |
| 7AAD | PerCP-Cy5.5 | BD Pharmingen 555815 |
| c-Kit | PE-Cy7 | ThermoFisher 25117182 |
| Sca-1 | V450 | BD Horizon 560653 |
| Cd16/32 | FITC | BD Pharmingen 561728 |

### Overexpression of dominant-negative RUNX1 binding domain

The dnRunx coding sequence was synthesized at Genscript and cloned into A reotiviral pMIG expression vector with XhoI and EcoRI. The introduced sequence is shown below:

ATGTACCCGTACGACGTTCCGGACTACGCTAGCGGCTCTGAGACACCTGGCAC
AAGCGAATCTGCCACACCTGAGAGCACCATGGTGGAGATCATCGCCGACCACC
CGGCCGAACTCGTCCGCACCGACAGCCCCAACTTCCTGTGCTCGGTGCTGCC
CTCGCACTGGCGCTGCAACAAGACCCTGCCCGTGGCCTTCAAGGTGGTAGCCC
TCGGAGAGGTACCAGATGGGACTGTGGTTACTGTCATGGCGGGTAACGATGAAA
ATTATTCTGCTGAGCTCCGGAATGCCTCTGCTGTTATGAAAAACCAAGTAGCAAG
GTTCAACGATCTGAGATTTGTGGGCCGGAGTGGACGAGGCAAGAGTTTCACCT
TGACCATAACCGTCTTCACAAATCCTCCCCAAGTAGCTACCTATCACAGAGCAAT
TAAAGTTACAGTAGATGGACCTCGGGAACCCAGAAGGCACAGACAGAAGCTTTA
A

Start codon | HA-tag | linker | RUNX binding domain (PMID 11431332) | Stop codon

## Detailed scRNA-seq computational analysis

Raw data were quantified against a genome-decoyed expanded (that is intronic) transcriptome (GENCODE vM25) as described: https://combine-lab.github.io/alevin-tutorial/2020/alevin-velocity/ with salmon-alevin (Srivastava et al. 2019). Spliced counts were then used for all downstream analysis in R and per-sample normalized using the deconvolution method from the Bioconductor package scran (computeSumFactors) (Lun et al. 2016) followed by batch rescaling with the batchelor package (multiBatchNorm). Samples were integrated with fastMNN from the batchelor package and clustered using Louvain based on the 1000 most variable genes. This revealed a clustering landscape confounded by cell cycle state (data not shown) indicated by two large groups of clustered with notably different expression of canonical cell cycle markers such as *Mki67* and *Top2a*. Differential genes between these two groups were identified using a t-test. These genes were then removed from the set of highly-variable genes to eliminate confounded clustering (genes were still eligible during differential expression analysis downstream of the clustering procedure), followed by a second round of dimensionality reduction and clustering, and removal of doublets (scDblFinder) and c-Kit$^{+}$ dendritic cells marked by high *Cd74* expression. Resulting clusters were annotated based on marker genes. Markers were defined as expressed by > 25% of cells per cluster and overexpressed on pseudobulk level (DESeq2, FDR < 0.05, absolute fold change > 1.5) in the given cluster versus all other clusters (Love et al. 2014). Differential expression between URE and WT clusters required expression by 10% of cells per cluster, FDR < 0.005 and absolute fold change > 1.5. Hierarchical clustering of DEGs in C1, C2 and C3 was carried out by the hclust function with Euclidean distance and ward.D2 agglomeration. Trajectory analysis was performed with slingshot, subsetting the dataset to C1 – C5 cells, forcing C1 to be the start- and both C4 and C5 to be the end points of the neutrophil- and monocyte trajectories. For visualization of lineage gene expression along the trajectory we used the C3 marker genes as neutrophil- and C5 markers (both from WT cells) as monocyte lineage markers. Cells were ordered in pseudotime and binned into 100 data points per cluster. For this, cells adjacent in pseudotime were averaged to achieve 100 bins. The per-bin gene expression score represented the median of the mean-subtracted lineage marker genes. Analysis of lineage bias in C1 cells was carried out by scoring

these cells with the neutrophil and monocyte lineage genes using the SingleR package.

### Assignment of differential peaks to signature genes from scRNA-seq

Differential accessibility analysis of ATAC-seq data was carried out with DESeq2 (FDR < 0.05, absolute fold change > 2). Differential peaks were assigned to signature 1-4 genes by the following criteria, under the assumption that scRNA-seq cluster C1 approximated LSKs and C3 approximated GMPs: Signature 1 was most expressed in WT C1 and least expressed in URE C3 so we connected it with differential ATAC-seq regions most accessible in WT LSKs and least accessible in URE GMPs. Signature 2 was most expressed in WT C3/C5 and least expressed in URE C1 so we connected it with differential ATAC-seq regions most accessible in WT GMPs and least accessible in URE LSKs. Signature 3 was most expressed in URE C1 and least expressed in WT C3 so we connected it with differential ATAC-seq regions most accessible in URE LSKs and least accessible in WT GMPs. Signature 4 was most expressed in URE C3 and least expressed in WT C1 so we connected it with differential ATAC-seq regions most accessible in URE GMPs and least accessible in WT LSKs. We only considered overlaps between the signature gene TSSs and ATAC-seq regions if located within 5kb up- or downstream of each other (10kb window). Peaks assigned to each signature were then scanned for motif enrichment with findMotifs.pl from Homer. As background we used a set of peaks consistently showing evidence against differential expression in all pairwise ATAC-seq comparisons. For this dedicated test against differential accessibility (in contrast to the default test <u>for</u> differential accessibility) we used DESeq2 with its “lessAbs” test, setting an upper fold change threshold of 1.75 and requiring an FDR < 0.05. In this test, low FDRs were interpreted as a surrogate that genomic regions were not differential. All tested contrasts were intersected ond regions significantly non-different were used to define the motif background set.

### Proteome mass spectrometry

A nanoflow HPLC (EASY-nLC1000, ThermoFisher) coupled online to a Q Exactive HF-X Hybrid Quadrupole-Orbitrap Mass Spectrometer (ThermoFisher) via a nano

electrospray ion source was utilized for the sample analysis. Approximately 500ng of peptides were loaded onto a 50-cm column with 75-μM diameter, packed in house with 1.9μM C18 ReproSil particles (Dr. Maisch GmbH). The column temperature was maintained using a homemade column oven at 60°C. The peptides were separated with the binary buffer system of 0.1% formic acid (buffer A) and 60% ACN plus 0.1% formic acid (buffer B), at a flow rate of 300nl/min. Peptides were eluted on a duration of 160 minutes with a gradient of 30% buffer B over 135 minutes and increased to 60% over for 20 minutes. This was followed by a quick ramp up to 95% over 5 minutes and declined back to 5% over 5' to re-equilibrate the column. Mass spectra was acquired in a data dependent mode. Briefly, with one full scan at a target of 3e6 ions (300-1650 m/z, R=60,000 at 200 m/z), followed by Top15 MS/MS scans with HCD (high energy collisional dissociation) (target 1e+5 ions, maximum filling time 28ms, Isolation window 1.4 m/z, and normalized collision energy 27), detected in the Orbitrap at a resolution of 15,000. Dynamic exclusion 30s and charge exclusion (unassigned, 1,6, -8 & >8) were enabled.

**shRNA screen**

A focused lentiviral shRNA dropout screen against signature 3 genes from the scRNA-seq analysis was performed in triplicates in Hox-WT, Hox-URE and URE-AML cells. shRNA-attached barcodes were used to identify shRNAs in genomic DNA (gDNA) after viral delivery. We included 344 of 346 signature 3 genes as these allowed us to design five unique shRNAs per gene. A total of 30 killing controls (Psma1, Rpl30, Polr2b with 10 barcodes each) and 45 non-targeting (luciferase) controls were added. The library was designed and delivered as a purified plasmid pool (pRSI17- U6-sh-UbiC-TagGFP2-2A-Puro) by Cellecta. Killing and negative controls were suggested by this company based on experience with these genes/barcodes in dropout screen across an extensive number of independent experiments. The library was delivered into target cells via lentivirus aided by RetroNectin (Takara). Following manufacturers recommendations, we aimed for an infection MOI of 0.3-0.5 to ensure that most cells harbor at most one viral integrate as well as for a library coverage/complexity > 200x the day after transfection and > 1000x at each time point that the cultured cells required splitting, all of which was measured and confirmed by FACS towards the GFP

in the screening plasmid. gDNA of 50% of cells was harvested as input control after 24h. Remaining cells were further cultured, selected with puromycin (concentration empirically determined per cell line in pilot experiments after empty vector transduction) and maintained until 10 cell divisions had happened (assessed by daily cell counting) followed by gDNA harvest. Illumina sequencing libraries towards the 22bp barcodes were created by a two-step nested PCR to first enrich the shRNA/barcode cassette from the gDNA followed by addition of adapter and index sequences for Illumina. For the nested PCR we followed suggestions from Cellecta towards creation of shRNA/sgRNA screen libraries (available at the company's website) using the primer pair "pRSI17_nested1_f/r" for round 1 and the "v2_Ad1.x/Ad2.x" dual-index Illumina primer pairs (all salt-free standard synthesis) for round 2. For sequencing we used the primers "pRSI17_custom_Read1/Index7/Index5" (all HPLC-purified synthesis). Libraries were sequenced as 22 bp single-end reads with 8bp dual indices on Nextseq 2000 using 10% PhiX as diversity spike-in. All shRNA and barcode sequences as well as primers are provided in Dataset EV3 and EV6. Reads were then aligned to the barcode reference using bowtie2 with the options "--end-to-end --very-sensitive --rdg 10000,10000 --rfg 10000,10000 --mp 10000,10000" to guarantee that only ungapped perfect matches were allowed to map. The count matrix was then created with featureCounts only considering perfect ungapped matches represented by a MAPQ score of 42. Counts were normalized to the NTC controls and analysed with MAGeCK to call "essential" genes. We required at least 3 out of 5 shRNAs to support this call, with a negative permutation FDR < 0.01. Fold changes between input libraries (assessment of potential infection bias) or "final" against input libraries were calculated with limma-voom and then quantile-normalized. Genes called as essential in either Hox-URE or URE-AML cells were further characterized to quantitatively identify preferential growth inhibition compared to WT cells. For this differential fold changes were obtained by subtracting the quantile-normalized log2 fold changes per shRNA of Hox-URE or URE-AML cells from Hox-WT.An empirical cutoff was defined as the 95$^{th}$ quantile of the DFCs of the non-targeting controls. At least 3 out of 5 shRNAs were required to show a DFC beyond this cutoff to define the preferential growth inhibition status.

## Appendix Figures

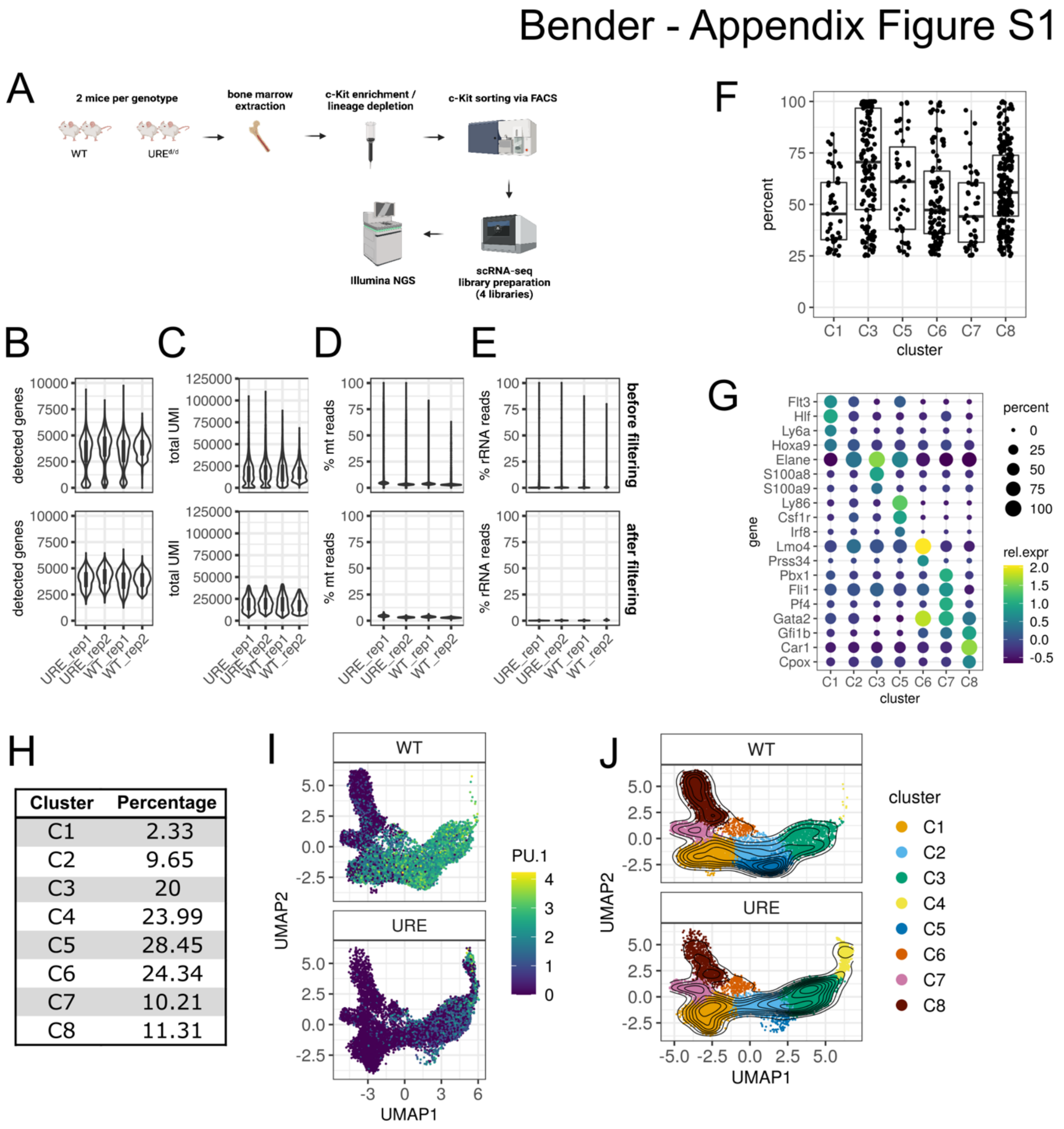


**Appendix Figure S1: scRNA-seq quality control and metrics.** A) Experimental layout. B-E) Summary of metrics used for quality control of scRNA-seq data before and after applying filter criteria (UMI=unique molecular identifiers, mt=mitochondrial). F) Percentage of cells per cluster expressing per-cluster marker genes. A hard cutoff of 25% was required for each gene to be eligible for marker detection. C2 was excluded as it did not show unique marker genes. A detailed summary towards percentage of expression was provided in Dataset EV1. G) Dotplot summarizing relative expression and percentage of expression per cluster for canonical marker

genes as highlighted in Figure 1B. H) Percentage of residual PU.1 gene expression in URE cells compared to WT per cluster. I/J) UMAP representation of the scRNA-seq dataset split by genotype and I) colored by PU.1 log2-expression or J) colored by cluster membership with contour lines representing cellular densities.

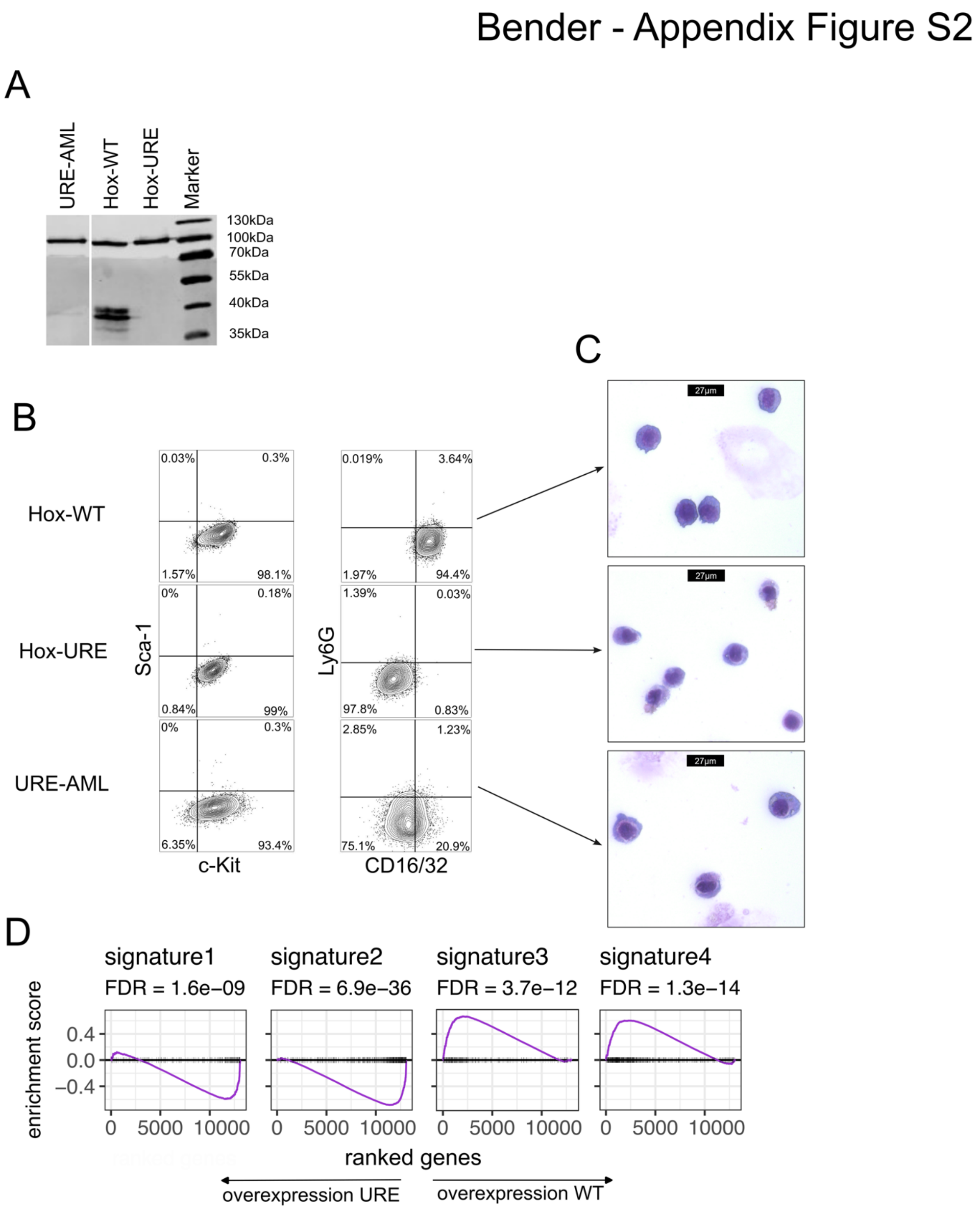


**Appendix Figure S2: Characterization of cell lines**. A) Western blot towards PU.1 with VCP as internal control for the three cell lines used in this study being a WT model (Hox-WT), a preleukemic URE$^{\Delta}$ (Hox-URE) and a leukemic blast model derived from a URE$^{\Delta}$ mouse (URE-AML). B) FACS analysis towards surface expression of c-Kit, Sca-1, CD16/32 and Ly6G. C) Morphological analysis with Wright-Giemsa staining of cytospin slides. D) Gene set enrichment analysis comparing Hox-URE vs Hox-WT

RNA-seq data towards the four transcriptional signatures identified in the scRNA-seq data (see Figure 2A). Permutation-based statistics were calculated using the R/Bioconductor package fgsea and corrected for multiple testing using Benjamini-Hochberg.

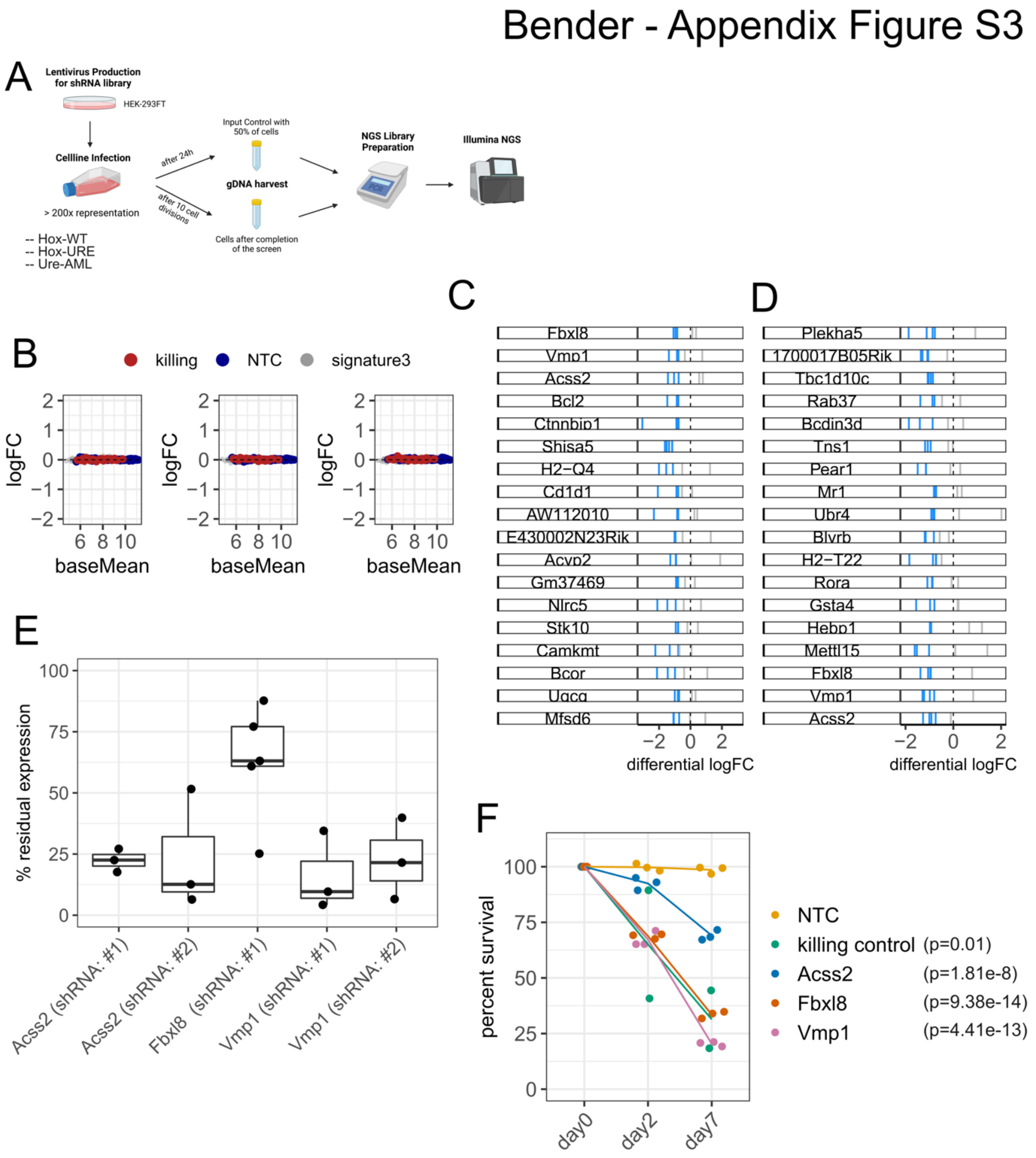


**Appendix Figure S3: shRNA screen of signature 3 genes.** A) Experimental layout. B) MA-plots indicating fold changes when contrasting individual input libraries to exclude a potential infection bias between cell lines. Plots indicated (from left to right) Hox-WT vs Hox-URE, Hox-WT vs URE-AML and Hox-URE vs URE-AML input libraries. C/D) Differential logFCs for shRNAs classified as more essential to Hox-URE vs Hox-WT (C) and URE-AML versus Hox-WT (D) cells. E) Assessment of shRNA knockdown efficiency by qPCR. URE-AML cells were retrovirally infected with two independent shRNAs per gene (one shRNA for Fbxl8) or non-targeting controls (NTC)

followed by gene expression measurement via RT-qPCR. Values indicated the percent remaining gene expression relative to the NTCs. F) Low-throughput validation of essential genes. URE-AML cells were transduced with individual shRNAs in a GFP expression plasmid followed by measurement of survival via FACS towards GFP after 2 and 7 days. Significances of stronger decay of survival over time for indicated genes compared to NTC were calculated by two-way ANOVA and adjusted for multiple testing by Benjamini-Hochberg (n=3 per day for all target genes except killing control which was n=2).

Bender - Appendix Figure S4

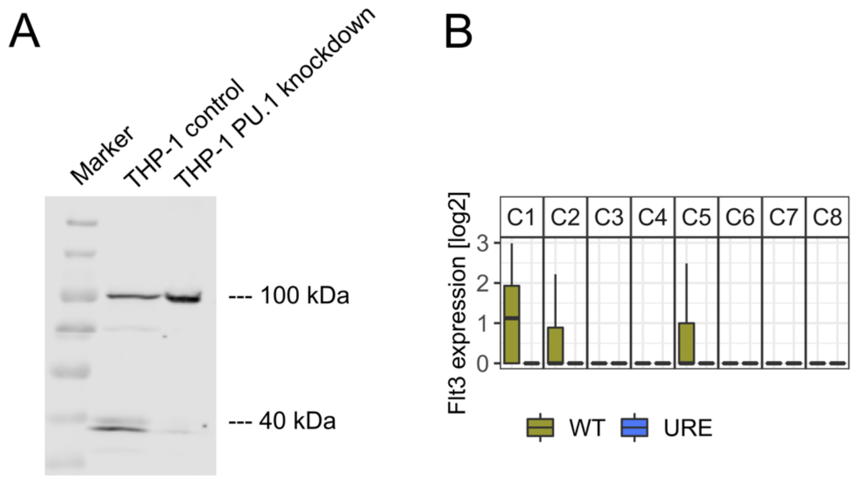


**Appendix Figure S4:** A) Western blot towards PU.1 (~40 kDa, Santa Cruz #sc-390405X, 1:1000) with a VCP loading control (~100 kDa, abcam #ab11433, 1:5000) in THP-1 with an shRNA-mediated PU.1 knockdown or non-targeting control cells. B) Expression of *Flt3* in the scRNA-seq dataset (single-cell resolution, ~10,000 cells in total).

Bender - Appendix Figure S5

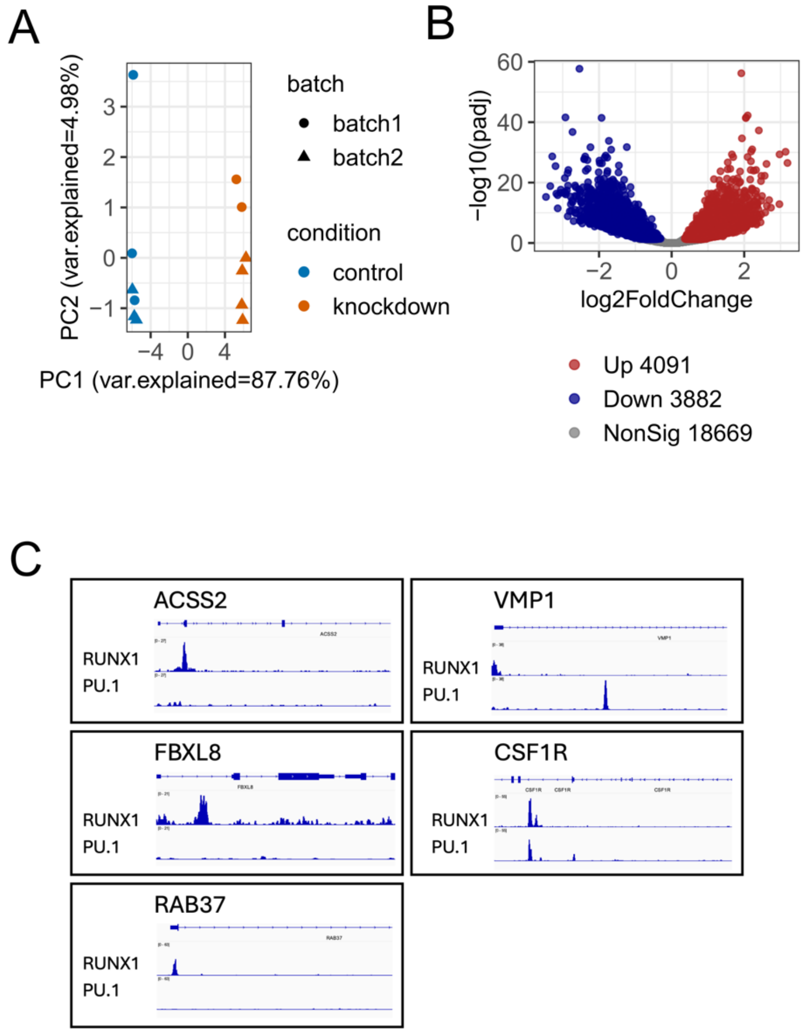


**Appendix Figure S5: RUNX1 ChIP-seq in THP-1 cells with either inducible PU.1 knockdown or scramble control.** A) Principal component analysis using the 500 most variable RUNX1 peaks. B) Volcano plot representing differential RUNX1 binding (FDR < 0.05, minimum absolute fold change 1.1, negative-binomial model using the R/Bioconductor package DESeq2) in PU.1 knockdown vs non-targeting control cells (n=6 cell line replicates per condition). C) Genomic browser tracks depicting promoter and promoter-proximal gene body regions at indicated genes towards RUNX1 in primary AML blasts (published ChIP-seq dataset GSE60130) and PU.1 in parental THP-1 cells (published ChIP-seq dataset GSE128834). Tracks per locus were scaled to the same maximum height. The CSF1R (M-CSF receptor) locus was included to demonstrate that absence of PU.1 in other loci was not due to a failed immunoprecipitation.

## Appendix References

Love, Michael I.; Huber, Wolfgang; Anders, Simon (2014): Moderated estimation of fold change and dispersion for RNA-seq data with DESeq2. In: *Genome biology* 15 (12), S. 550. DOI: 10.1186/s13059-014-0550-8.

Lun, Aaron T. L.; Bach, Karsten; Marioni, John C. (2016): Pooling across cells to normalize single-cell RNA sequencing data with many zero counts. In: *Genome biology* 17, S. 75. DOI: 10.1186/s13059-016-0947-7.

Srivastava, Avi; Malik, Laraib; Smith, Tom; Sudbery, Ian; Patro, Rob (2019): Alevin efficiently estimates accurate gene abundances from dscRNA-seq data. In: *Genome biology* 20 (1), S. 65. DOI: 10.1186/s13059-019-1670-y.
