# Supplementary material for: Redistribution of PU.1 partner transcription factor RUNX1 binding secures cell survival during leukemogenesis
Source: EMBO J. 2024 Nov 14;43(24):6291–309. doi: 10.1038/s44318-024-00295-y (PMC11649769; doi:10.1038/s44318-024-00295-y)
Supplement: Supplementary file 14 — Source data Fig. 5 [file 44318_2024_295_MOESM14_ESM.zip › Source_Data_Figure_5/source_Data_figure_5c_blot.pptx]

## Slide 1
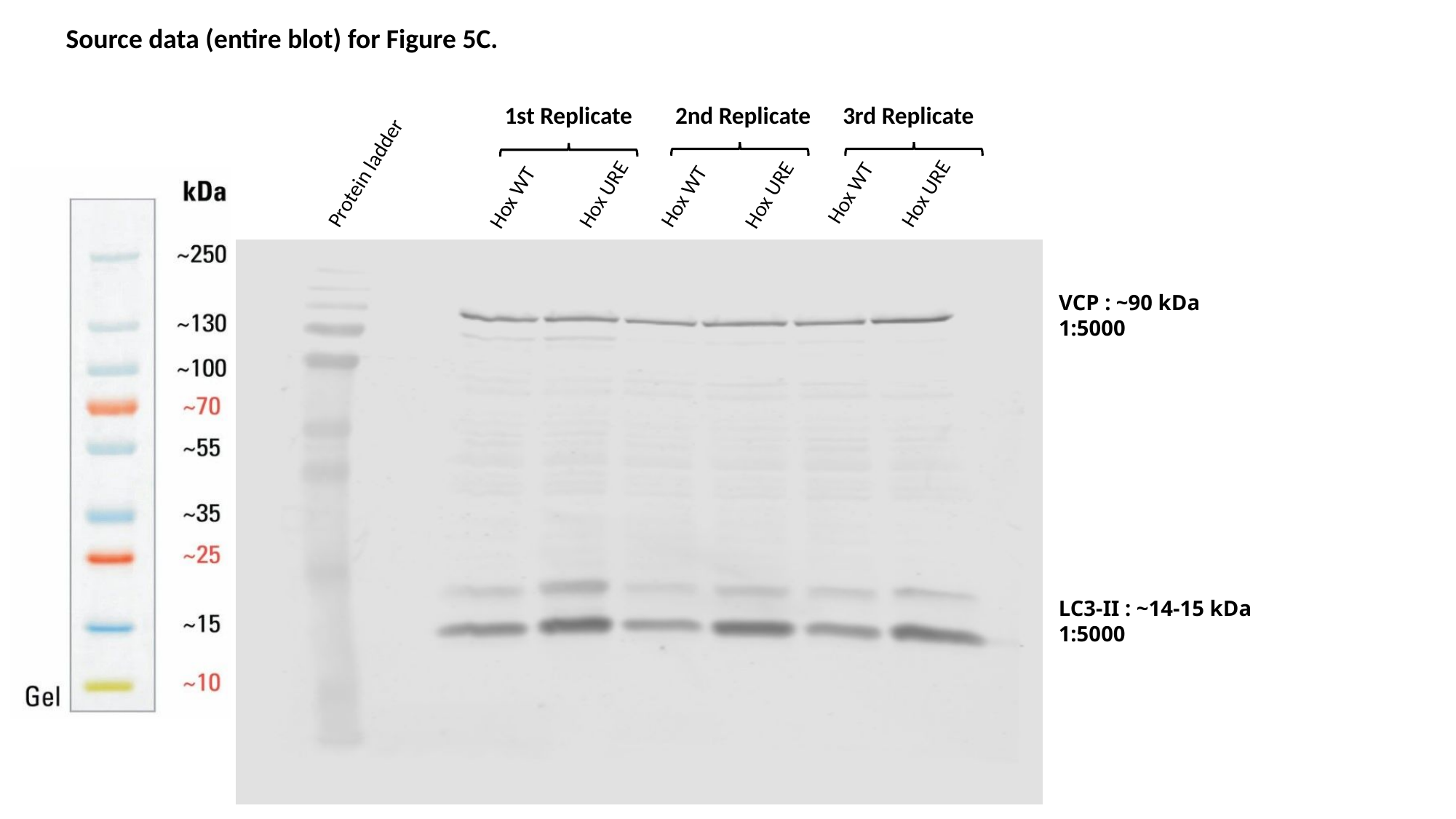

Source data (entire blot) for Figure 5C.
1st Replicate
2nd Replicate
3rd Replicate
Hox WT
Protein ladder
Hox URE
Hox URE
Hox URE
Hox WT
Hox WT
VCP : ~90 kDa
1:5000
LC3-II : ~14-15 kDa
1:5000
