# Supplementary material for: Redistribution of PU.1 partner transcription factor RUNX1 binding secures cell survival during leukemogenesis
Source: EMBO J. 2024 Nov 14;43(24):6291–309. doi: 10.1038/s44318-024-00295-y (PMC11649769; doi:10.1038/s44318-024-00295-y)
Supplement: Supplementary file 16 — Appendix Figure Source Data [file 44318_2024_295_MOESM16_ESM.zip › Source_Data_Supplemental/source_data_figure_appendix_2a.pptx]

## Slide 1
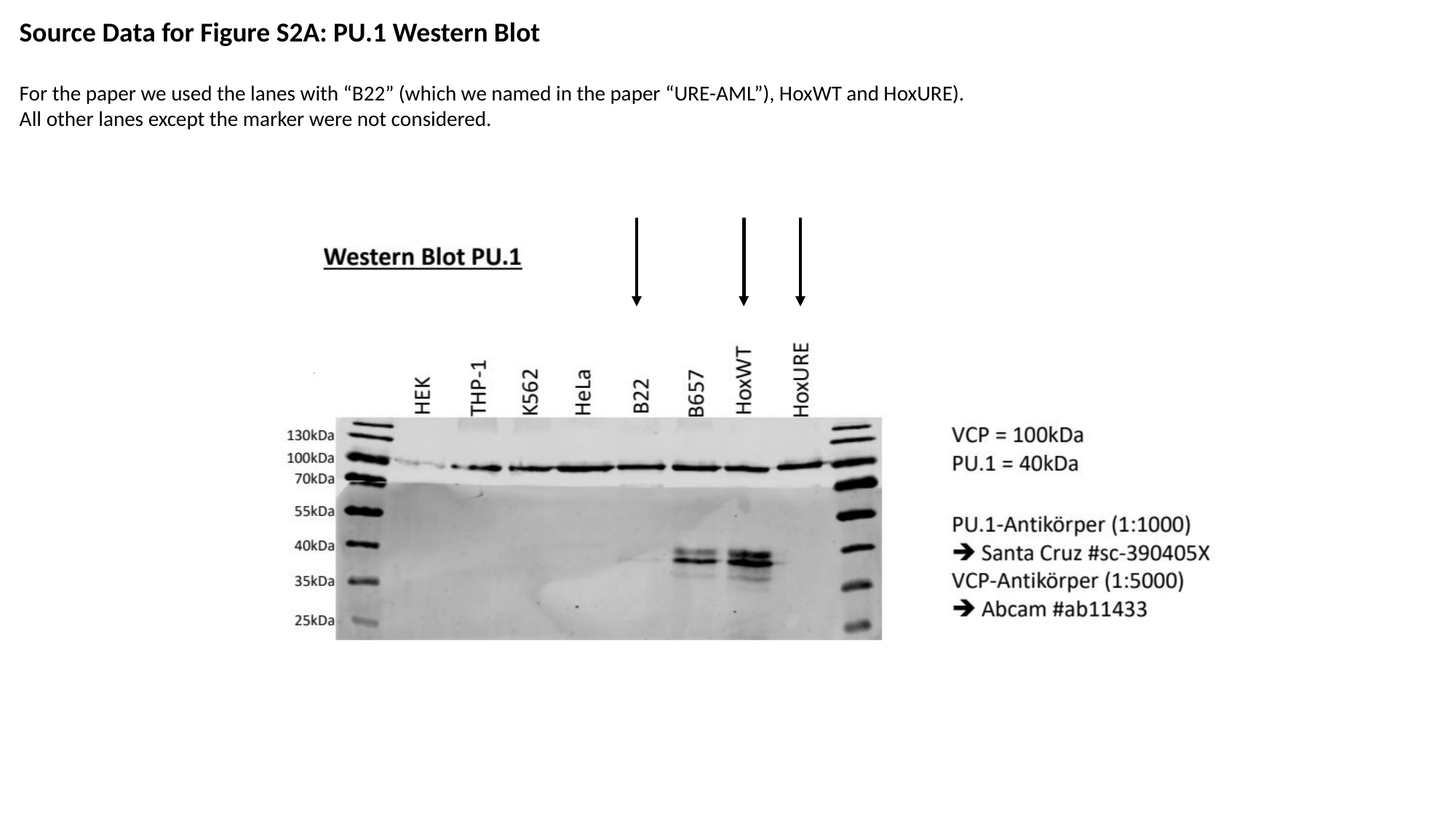

Source Data for Figure S2A: PU.1 Western Blot
For the paper we used the lanes with “B22” (which we named in the paper “URE-AML”), HoxWT and HoxURE).
All other lanes except the marker were not considered.
