# Supplementary material for: Redistribution of PU.1 partner transcription factor RUNX1 binding secures cell survival during leukemogenesis
Source: EMBO J. 2024 Nov 14;43(24):6291–309. doi: 10.1038/s44318-024-00295-y (PMC11649769; doi:10.1038/s44318-024-00295-y)
Supplement: Supplementary file 16 — Appendix Figure Source Data [file 44318_2024_295_MOESM16_ESM.zip › Source_Data_Supplemental/source_data_figure_appendix_4a.pptx]

## Slide 1
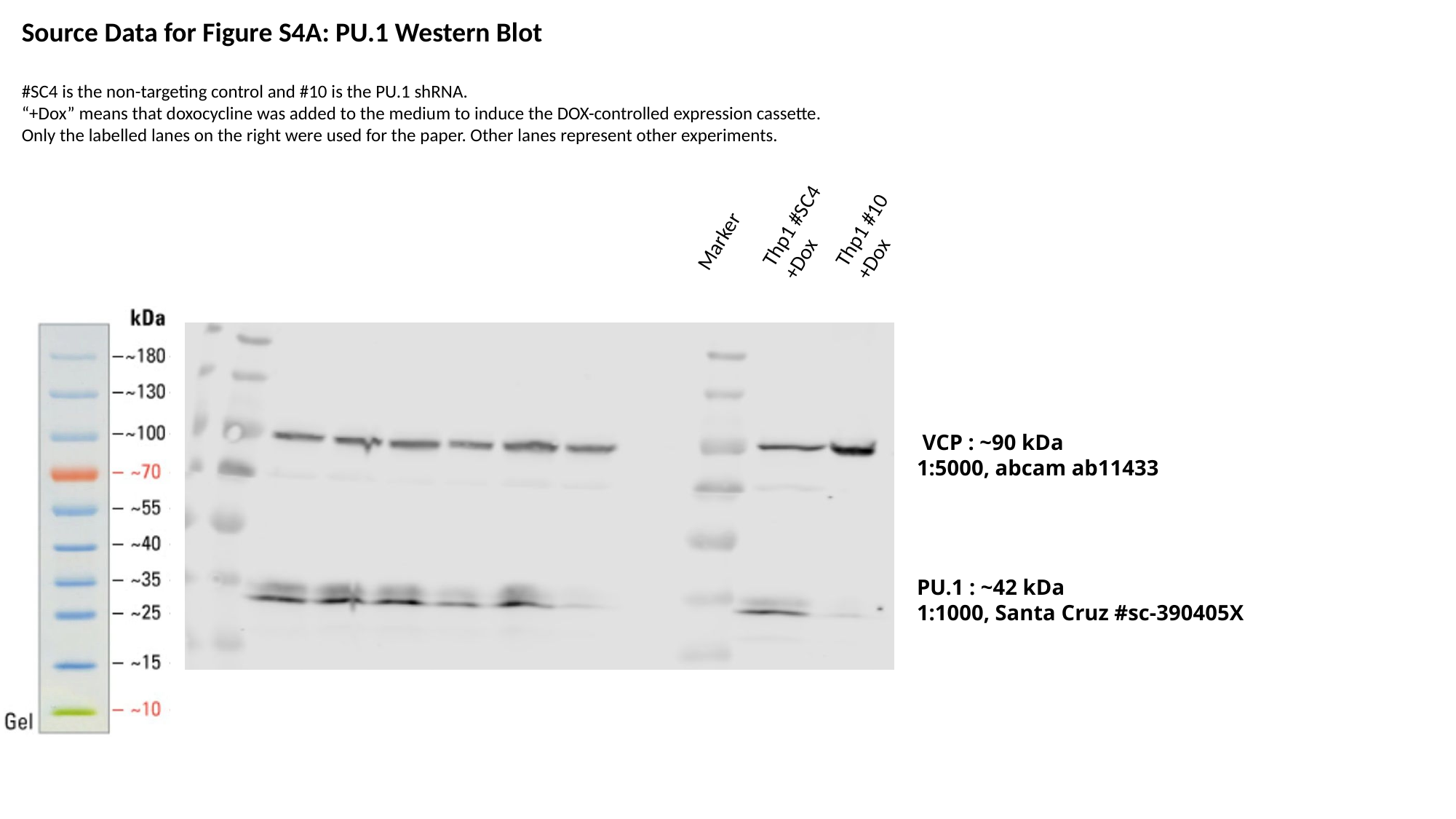

Source Data for Figure S4A: PU.1 Western Blot
#SC4 is the non-targeting control and #10 is the PU.1 shRNA.
“+Dox” means that doxocycline was added to the medium to induce the DOX-controlled expression cassette.
Only the labelled lanes on the right were used for the paper. Other lanes represent other experiments.
Thp1 #SC4
+Dox
Thp1 #10
+Dox
Marker
 VCP : ~90 kDa
1:5000, abcam ab11433
PU.1 : ~42 kDa
1:1000, Santa Cruz #sc-390405X
